# Diathermy-induced Ventricular Fibrillation

**DOI:** 10.19102/icrm.2021.120203

**Published:** 2021-02-15

**Authors:** Sanoj Chacko, Saud B. Haseeb, Sohaib Haseeb, Joseph DeBono, Howard Marshall

**Affiliations:** ^1^Heart Rhythm Service, Queen’s University, Kingston, Ontario, Canada; ^2^Faculty of Health Sciences, McMaster University, Hamilton, Ontario, Canada; ^3^Queen Elizabeth Hospital, Mindelsohn Way, Birmingham B15 2TH, UK

**Keywords:** Defibrillation, diathermy, ventricular fibrillation

## Abstract

Diathermy is extensively used in patients for intracardiac device implant and extraction. While diathermy helps with adequate hemostasis, it may rarely be associated with fatal dysrhythmias. We report a case of diathermy-induced ventricular fibrillation during device extraction. The case highlights the importance and supports the involvement of a defibrillation facility during pacemaker revisions requiring diathermy.

## Introduction

Surgical diathermy involves the use of a high-frequency electric current in the cutting and coagulation of body tissue. In cardiology, monopolar diathermy is extensively deployed in patients to support permanent pacemaker (PPM) implantation and extraction. While diathermy helps with adequate hemostasis and reduces bleeding complications, it may rarely be associated with fatal dysrhythmias. We report a case of diathermy-induced ventricular fibrillation (VF) during device extraction.

## Case presentation

**Figure 1:**
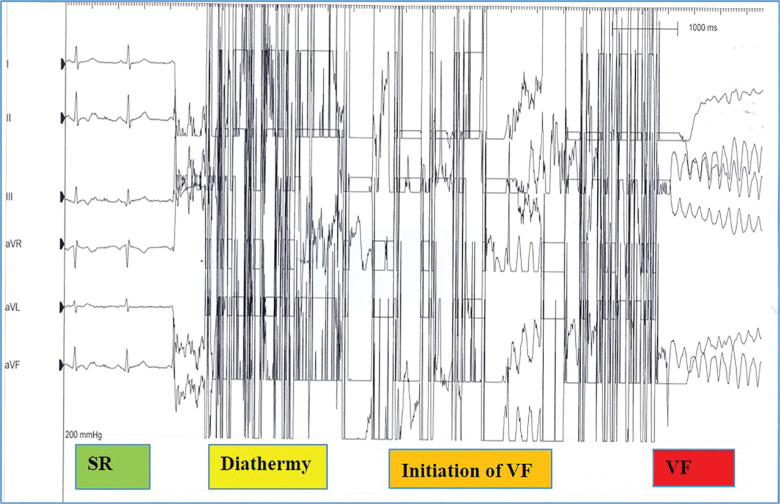
At the beginning, sinus rhythm can be seen; however, the application of diathermy triggered VF. SR: sinus rhythm; VF: ventricular fibrillation.

A 44-year-old man was transferred from his local hospital to undergo pacemaker extraction following the emergence of an infection in the pacemaker system. He had received a Sensia SEDRO1 dual-chamber PPM (Medtronic, Minneapolis, MN, USA) 25 years ago that was implanted for sinus node dysfunction and syncope. He had no other significant comorbidities but showed evidence of a pacemaker pocket infection with device erosion. His blood cultures were negative and he was systemically well with stable hemodynamics and without the need for any inotropic support. A preprocedure transesophageal echocardiogram confirmed a structurally normal heart. A pacemaker check revealed an underlying sinus rhythm at 70 bpm. Based on the above, he had a class 1 indication for device extraction and the procedure was performed under sedation. Prior to extraction, his pacemaker was programmed to VVI, bipolar mode, defibrillation pads were connected, and continuous rhythm and hemodynamic monitoring were performed. Using an electrosurgery generator (CONMED, Utica, NY, USA) for monopolar diathermy, an incision was made over the pacemaker pocket in an attempt to extract the generator. The pacing wires were not exposed at this point and were located beneath the pacemaker generator without no contact with the diathermy blade. During diathermy, VF was noted, with loss of cardiac output. The patient was immediately cardioverted to sinus rhythm successfully with a single 150-joule direct-current shock **(Figure 1)**. The patient subsequently tolerated the remainder of the procedure very well and experienced an uncomplicated device extraction.

## Discussion

The rate of PPM implantation and its prevalence continue to rise with the expanding aging population worldwide.^1^ As a result, in addition to the ongoing increase in device implantation rates, there is also a growing need for device replacements either due to battery depletion or, rarely, device infections.^2^ Diathermy is extensively used and is very effective in maintaining good hemostasis in patients who are on anticoagulation or antiplatelet therapy. It is well-recognized that the use of diathermy can cause interference, resulting in pacing inhibition and asystole in patients who are pacing-dependent. However, the occurrence of ventricular arrhythmia is rare and the mechanism behind it in this context remains unclear, particularly in patients with a structurally normal heart.

Electromagnetic devices, such as PPMs and implantable cardioverter-defibrillators (ICDs), have been reported to interfere with electrosurgical devices.^3^ In this context, the interference caused by diathermy can lead to potentially life-threatening arrhythmias. A rare case of diathermy-induced VF was previously reported as a result of an insulation defect in the high-voltage lead at the level of the pectoral muscles.^4^ In our case, a possible explanation for the VF could be due to the close proximity between the diathermy blade and the PPM generator. Diathermy usage in patients with either a pacemaker or an ICD can result in electrical interference that may initiate inappropriate shock, inhibition, reprograming, or damage to the device.^5–7^ Preventative strategies include programing the PPM to asynchronous VOO or DOO mode (ie, pulse generator delivers a pacing stimulus at a fixed rate without sensing capabilities), application of a magnet over the device, disabling of the ICD therapy, the use of short bursts if monopolar diathermy is unavoidable, placement of the dispersive electrode in a position as far as possible from the cardiac device, and electrocardiogram monitoring for PPM in the case of inhibition.

In addition to adhering to the general safety principles of diathermy, the present case also highlights the importance of involving a defibrillation facility, which should be readily available when necessary, and supports the standard practice of using defibrillation pads as a safety net in all patients who require diathermy.
